# Characterization of Fine Particulate Matter and Associations between Particulate Chemical Constituents and Mortality in Seoul, Korea

**DOI:** 10.1289/ehp.1104316

**Published:** 2012-03-22

**Authors:** Ji-Young Son, Jong-Tae Lee, Ki-Hyun Kim, Kweon Jung, Michelle L. Bell

**Affiliations:** 1 School of Forestry and Environmental Studies, Yale University, New Haven, Connecticut, USA; 2 Department of Environmental Health, College of Health Science, Korea University, Seoul, Korea; 3 Department of Environment and Energy, Sejong University, Seoul, Korea; 4 Seoul Metropolitan Institute of Public Health and Environment, Seoul, Korea

**Keywords:** chemical constituents, mortality, PM_2.5_, time-series

## Abstract

Background: Numerous studies have linked fine particles [≤ 2.5 µm in aerodynamic diameter (PM_2.5_)] and health. Most studies focused on the total mass of the particles, although the chemical composition of the particles varies substantially. Which chemical components of fine particles that are the most harmful is not well understood, and research on the chemical composition of PM_2.5_ and the components that are the most harmful is particularly limited in Asia.

Objectives: We characterized PM_2.5_ chemical composition and estimated the effects of cause-specific mortality of PM_2.5_ mass and constituents in Seoul, Korea. We compared the chemical composition of particles to those of the eastern and western United States.

Methods: We examined temporal variability of PM_2.5_ mass and its composition using hourly data. We applied an overdispersed Poisson generalized linear model, adjusting for time, day of week, temperature, and relative humidity to investigate the association between risk of mortality and PM_2.5_ mass and its constituents in Seoul, Korea, for August 2008 through October 2009.

Results: PM_2.5_ and chemical components exhibited temporal patterns by time of day and season. The chemical characteristics of Seoul’s PM_2.5_ were more similar to PM_2.5_ found in the western United States than in the eastern United States. Seoul’s PM_2.5_ had lower sulfate (SO_4_) contributions and higher nitrate (NO_3_) contributions than that of the eastern United States, although overall PM_2.5_ levels in Seoul were higher than in the United States. An interquartile range (IQR) increase in magnesium (Mg) (0.05 μg/m^3^) was associated with a 1.4% increase (95% confidence interval: 0.2%, 2.6%) in total mortality on the following day. Several components that were among the largest contributors to PM_2.5_ total mass—NO_3_, SO_4_, and ammonium (NH_4_)—were moderately associated with same-day cardiovascular mortality at the *p* < 0.10 level. Other components with smaller mass contributions [Mg and chlorine (Cl)] exhibited moderate associations with respiratory mortality on the following day (*p* < 0.10).

Conclusions: Our findings link PM_2.5_ constituents with mortality and have implications for policy making on sources of PM_2.5_ and on the relevance of PM_2.5_ health studies from other areas to this region.

Many epidemiological studies have provided evidence of adverse health effects of particulate matter, including particulate matter (PM) ≤ 2.5 μm in aerodynamic diameter (PM_2.5_) (Dockery et al. 1993; Franklin et al. 2007; Pope and Dockery 2006; Schwartz et al. 1996). However, most of these studies and the regulations designed to protect public health from airborne particles have focused on the risk associated with the total mass of particles, without regard to the particle characteristics other than size. Studies show that the chemical composition of particles exhibits substantial variation spatially and temporally (Bell et al. 2007; Zanobetti et al. 2009). Identifying which chemical components of particles are most harmful was identified as a critical research need by several agencies, including the U.S. Environmental Protection Agency (U.S. EPA 2009), the Health Effects Institute (2004), and committees of the National Research Council (2004). Understanding and characterizing the health effects of PM components and sources is crucial for effective regulatory control of particulate matter pollution.

Several studies have estimated risks based on the differential toxicity of various sources and components of the PM_2.5_ mixture. Laden et al. (2000) identified several distinct source-related fractions of fine particles and examined the association of these fractions with daily mortality in six U.S. cities. Peng et al. (2009) investigated the association between hospital admissions for cardiovascular and respiratory disease and chemical components of PM_2.5_ in the United States. Bell et al. (2009) also reported links between geographical and seasonal differences in the estimated short-term effects of PM_2.5_ on the hospitalizations and variations in PM_2.5_ chemical composition. Recent analyses have investigated individual U.S. locations, including study of the effects of PM_2.5_ components on daily mortality for Seattle, Washington, and Detroit, Michigan (Zhou et al. 2011), and on hospitalizations and mortality in New York, New York (Ito et al. 2011). A source apportionment analysis for risk of hospital admissions was conducted for Manhattan, New York (Lall et al. 2011). Previous studies reported mortality associations for several chemical components of PM_2.5_, including major particulate components such as elemental carbon (EC), organic carbon (OC), sulfate (SO_4_), and nitrate (NO_3_) as well as trace components like nickel (Ni) and arsenic (As) (Burnett et al. 2000; Franklin et al. 2008; Ostro et al. 2007).

The vast majority of studies examining how the chemical structure of particles relates to health were conducted in North America and Europe. Because the composition and sources of particles vary dramatically by location, studies are needed in other regions.

In Korea, research on the adverse effects of PM_2.5_ is limited, especially in relation to specific types of particles. To date, several studies have been conducted in Korea to explore the health impacts of PM_2.5_ including mortality (Cho et al. 2008; Kang et al. 2006) and lung function (Lee et al. 2007a; Yu et al. 2007). Most of these studies, however, focused on the total mass of a given size of particles. The specific sources and constituents responsible for the adverse effects of PM_2.5_ have not been investigated in this region, although a few studies have considered PM metals in relation to oxidative stress (Bae et al. 2010) or to lung function (Hong et al. 2007). Thus, relatively little is known about the chemical composition of PM_2.5_ in Korea and how different types of particles may impact public health.

Our study had two main goals. The first was to characterize the chemical composition of PM_2.5_ in Seoul, Korea. We were able to characterize the daily pattern of pollutant levels using the concentrations of PM_2.5_ components acquired at hourly intervals. This approach differs from most of the previous work, such as in the United States and Europe, which has focused on daily (24-hr) values (e.g., Bell et al. 2010; Halonen et al. 2009; Ito et al. 2011; Ostro et al. 2011). The hourly values allow exploration of daytime exposure that may better reflect personal exposure than daily values. The second aim was to estimate the cause-specific mortality effects of PM_2.5_ mass and its constituents in Seoul, Korea. Although the timeframe of our study was shorter than that of other comparable studies, our exposure data were available every day rather than the every three to six days as is the case for most previous studies. Daily data also allowed us to estimate the effects for cumulative lags. To the best of our knowledge, no previous study has examined the mortality impacts of PM_2.5_ chemical constituents in Korea or elsewhere in Asia.

## Methods

*Data.* Neither PM_2.5_ total mass nor PM_2.5_ chemical components are routinely measured in South Korea, although efforts are underway to expand the monitoring network for PM_2.5_ total mass [Ministry of Environment, Korea (MOE) 2011]. We collected hourly air samples from the Gwangjin monitoring station (~ 10.3 m above ground, 37.32°N, 127.05°E) in Seoul, Korea, from August 2008 to October 2009. This site is surrounded by commercial and residential buildings, and is approximately several hundred meters from the main road. Samples were collected using the ADI2080 ambient air monitoring system (Marga; Metrohm Applikon, Schiedam, the Netherlands). This system allows monitoring of ionic components of PM_2.5_ along with several gaseous components with automatic flow control by a vacuum pump. Ionic species in gas phase is first collected into absorption solution, as they diffuse through the wetted rotating denuder. Aerosol particles that are not caught by the solution are dissolved in the form of supersaturated vapor by the steam jet aerosol collector. Analysis of gaseous and particulate phase components (and standards) is carried out by transferring 25 mL of solution per hour to the ion chromatography. The monitoring system produces hourly estimates of PM_2.5_ total mass, and PM_2.5_ levels of OC; EC; major cations of calcium (Ca), sodium (Na), potassium (K), magnesium (Mg), and ammonium (NH_4_); and major anions of chlorine (Cl), sulfate (SO_4_), and nitrate (NO_3_). We examined whether hourly reported values were below the detection limit (BDL) for each component, and reported the percentage of data with BDL observations. For each component, hourly BDL data were replaced by half of the detection limit values for the analysis, and two sensitivity analyses were performed by using the recorded values and by omitting BDL values. The hourly values were averaged over two periods: *a*) daytime exposure (0800–2000 hours) and *b*) 24-hr exposure.

Daily meteorological data for Seoul during the study period were calculated based on hourly ambient temperature, hourly relative humidity, and 3-hr barometric pressure data acquired from the Korea Meteorological Administration (KMA; Seoul, Korea). Daily death counts in Seoul for the study period were obtained from the National Statistical Office, Republic of Korea (Daejeon, Korea). We classified mortality data into all causes of death [*International Classification of Diseases*, *10th Revision* (ICD-10; codes A00–R99), cardiovascular causes (codes I00–I99), and respiratory causes (codes J00–J99)] (World Health Organization 2007); external causes of death (e.g., accidents) were not considered. Analyses were stratified by cause of death.

*Statistical analysis.* First, we characterized the chemical composition of PM_2.5_ and examined the temporal variability of PM_2.5_ composition by different temporal intervals: year, season, day, and hour. We identified relative contribution of each component to total PM_2.5_ mass and then calculated correlations between PM_2.5_ total mass and each component. Seasons were defined based on 3-month periods; for example, summer was defined as June through August. Days during Asian dust storms were compared with days without storms.

To estimate the relationship between daily mortality and PM_2.5_ mass and chemical constituents, we applied an overdispersed Poisson generalized linear model with natural cubic splines for time and meteorology.

ln[E(Y*_t_*)]= β*^^j^^*__0__ + β*^^j^^*Χ*^^j^^__t__* + a*^^j^^*DOW*__t__* + ns(time*__t__*) + ns(temperature*__t__*) + ns(humidity*__t__*), [1]

where E(Y*_t_*) is the expected number of deaths on day *t*; β*^^j^^*__0__ is the model intercept for exposure *j* (i.e., PM_2.5_ total mass or a particular chemical component); a*^j^* is the vector of regression coefficients for day of the week for model of exposure *j*; DOW*_t_* is the categorical variable for day of the week; ns(time*_t_*) is the natural cubic spline of a variable representing time to adjust for long-term trends and seasonality, with 6 degrees of freedom (df) per year; ns(temperature*_t_*) is the natural cubic spline of current-day temperature on day *t*, with 3 df; and ns(humidity*_t_*) is the natural cubic spline of current-day humidity on day *t*, with 3 df. The variable Χ*^^j^^__t__* represents the level of exposure *j* on day *t*, where the exposure is PM_2.5_ total mass or a specific component. The variable β*^^j^^* denotes the relationship between exposure *j* and mortality risk. Each exposure and cause of mortality was modeled separately. Similar models have been used in recent research of PM_2.5_ chemical components and mortality in other regions (e.g., Zhou et al. 2011).

We considered PM_2.5_ total mass with lag structures of exposure on the same day (lag 0) and up to 3 days before (lag 0, lag 1, lag 2, and lag 3) and cumulative lags (lag 0–1, lag 0–2, and lag 0–3). We selected the lag with the most certain effect estimates (largest *t*-statistics) for each cause of death for subsequent analysis on PM_2.5_ chemical components. All analyses were conducted using R 2.10.1 (R Foundation for Statistical Computing, Vienna, Austria). Results are expressed as the percentage change in mortality with 95% confidence interval (CI) per interquartile range (IQR) increase of PM_2.5_ mass and each PM_2.5_ chemical constituent.

## Results

*Characterization of PM_2.5_ chemical composition.* Table 1 summarizes the data on PM_2.5_ mass and chemical components and the contribution of each component to PM_2.5_ total mass for the entire period and by season. The total number of observations for each component was 10,968 (equivalent to hourly data for 457 days). Some values were BDL, especially for Na and Mg. The mean ± SD PM_2.5_ concentration was 26.6 ± 16.5 μg/m^3^ for the entire period (August 2008 through October 2009). PM_2.5_ levels had seasonal patterns, with higher values in winter (December through February) (34.4 ± 20.2 μg/m^3^) and lower values in summer (21.3 ± 11.7 μg/m^3^). Daytime and 24-hr averages for the entire period were similar, with slightly higher values in the 24-hr average, for PM_2.5_ mass and for each chemical component [see Supplemental Materials, Figure 1 (http://dx.doi.org/10.1289/ehp.1104316)]. For example, the average 24-hr values for PM_2.5_ total mass, OC, and EC were 26.6, 5.7, and 2.2 μg/m^3^ compared with their respective average daytime concentrations (25.0, 5.4, and 2.0 μg/m^3^, respectively).

**Table 1:** Summary statistics for PM_2.5_ mass and chemical component concentrations in Seoul, Korea, (August 2008 through October 2009).

Components	Mass of component (μg/m3)					Average percentage of PM2.5 total mass (IQR of percent)
	Average ± SD	Minimum–Maximum	IQR			
Entire period								
PM2.5		26.6 ± 16.5		3.7–102.4		16.0		—
OC		5.7 ± 2.9		0.9–16.0		3.4		23.4 (6.3)
EC		2.2 ± 1.3		0.4–8.2		1.7		8.3 (3.2)
Cl		0.3 ± 0.4		0.0–2.5		0.2		1.2 (1.1)
NO3		4.4 ± 3.1		0.4–19.3		3.3		16.3 (6.3)
SO4		4.3 ± 3.6		0.0–25.1		3.2		15.5 (6.9)
Na		0.1 ± 0.1		0.0–0.8		0.1		0.6 (0.7)
NH4		2.6 ± 2.2		0.1–15.3		2.0		8.6 (2.7)
K		0.2 ± 0.2		0.0–1.0		0.2		1.0 (0.8)
Mg		0.1 ± 0.1		0.0–0.4		0.05		0.3 (0.3)
Ca		0.2 ± 0.1		0.0–0.9		0.2		1.2 (1.1)
Spring (March through May)								
PM2.5		28.8 ± 14.4		6.7–78.0		18.3		—
OC		5.8 ± 2.5		1.8–13.0		3.5		21.6 (6.2)
EC		2.0 ± 1.2		0.4–6.2		1.6		6.8 (2.1)
Cl		0.2 ± 0.2		0.0–1.1		0.2		0.7 (0.7)
NO3		4.6 ± 2.0		1.4–10.7		2.6		16.9 (4.9)
SO4		4.0 ± 2.9		0.4–15.5		2.5		13.0 (4.7)
Na		0.0 ± 0.0		0.0–0.2		0.0		0.1 (0.1)
NH4		2.5 ± 1.6		0.3–8.5		1.8		8.0 (1.3)
K		0.2 ± 0.1		0.0–0.7		0.2		0.7 (0.4)
Mg		0.1 ± 0.0		0.0–0.1		0.0		0.3 (0.2)
Ca		0.2 ± 0.1		0.0–0.7		0.1		1.2 (0.9)
Summer (June through August)								
PM2.5		21.3 ± 11.7		4.2–63.5		17.3		—
OC		4.5 ± 2.2		0.9–12.6		2.8		23.2 (8.1)
EC		1.7 ± 0.9		0.4–5.2		1.4		8.5 (3.6)
Cl		0.1 ± 0.1		0.0–0.3		0.1		0.7 (0.5)
NO3		2.9 ± 2.0		0.4–9.4		3.0		13.6 (7.0)
SO4		3.6 ± 2.5		0.0–13.1		3.2		16.2 (7.4)
Na		0.1 ± 0.1		0.0–0.6		0.1		0.7 (0.7)
NH4		1.9 ± 1.3		0.1–5.7		1.7		7.6 (2.9)
K		0.1 ± 0.1		0.0–0.5		0.1		0.6 (0.6)
Mg		0.1 ± 0.1		0.0–0.4		0.1		0.6 (0.5)
Ca		0.3 ± 0.1		0.1–0.5		0.1		1.8 (1.1)
Autumn (September through November)								
PM2.5		24.9 ± 16.8		3.7–95.3		15.0		—
OC		5.3 ± 2.7		1.2–14.4		3.0		23.5 (5.7)
EC		2.1 ± 1.2		0.4–6.1		1.5		8.8 (3.6)
Cl		0.2 ± 0.1		0.0–0.4		0.1		1.0 (0.7)
NO3		4.4 ± 3.5		0.5–19.3		3.3		17.3 (6.4)
SO4		4.7 ± 4.4		0.4–25.1		4.4		17.1 (8.2)
Na		0.1 ± 0.1		0.0–0.4		0.1		0.7 (0.7)
NH4		2.8 ± 2.6		0.2–15.3		2.2		9.8 (4.9)
K		0.2 ± 0.1		0.0–0.4		0.1		1.1 (0.7)
Mg		0.0 ± 0.0		0.0–0.2		0.1		0.3 (0.3)
Ca		0.2 ± 0.1		0.0–0.5		0.1		1.1 (1.0)
Winter (December through February)								
PM2.5		34.4 ± 20.2		9.4–102.4		21.2		—
OC		8.0 ± 3.5		2.6–16.0		5.3		25.4 (5.0)
EC		3.0 ± 1.8		0.5–8.2		2.6		8.6 (2.5)
Cl		0.9 ± 0.6		0.1–2.5		0.9		2.6 (1.6)
NO3		6.0 ± 3.7		0.9–16.7		4.2		17.5 (4.8)
SO4		4.9 ± 3.9		1.2–20.8		3.0		14.4 (5.0)
Na		0.1 ± 0.1		0.0–0.8		0.2		0.5 (0.6)
NH4		3.3 ± 2.5		0.1–12.5		2.6		8.8 (2.9)
K		0.4 ± 0.2		0.0–1.0		0.3		1.4 (0.7)
Mg		0.0 ± 0.0		0.0–0.1		0.0		0.1 (0.1)
Ca		0.1 ± 0.1		0.0–0.9		0.1		0.6 (0.7)
—, not applicable. Calculations were based on daily values using hourly observations. Percentages of BDL observations for each pollutant: PM2.5, 0.0; OC, 0.0; EC, 0.3; Cl, 15.7; NO3, 0.2; SO4, 0.8; Na, 43.4; NH4, 0.6; K, 13.8; Mg, 28.6; Ca, 6.6. BDL data were replaced by half of the detection limit values.								

**Figure 1:**
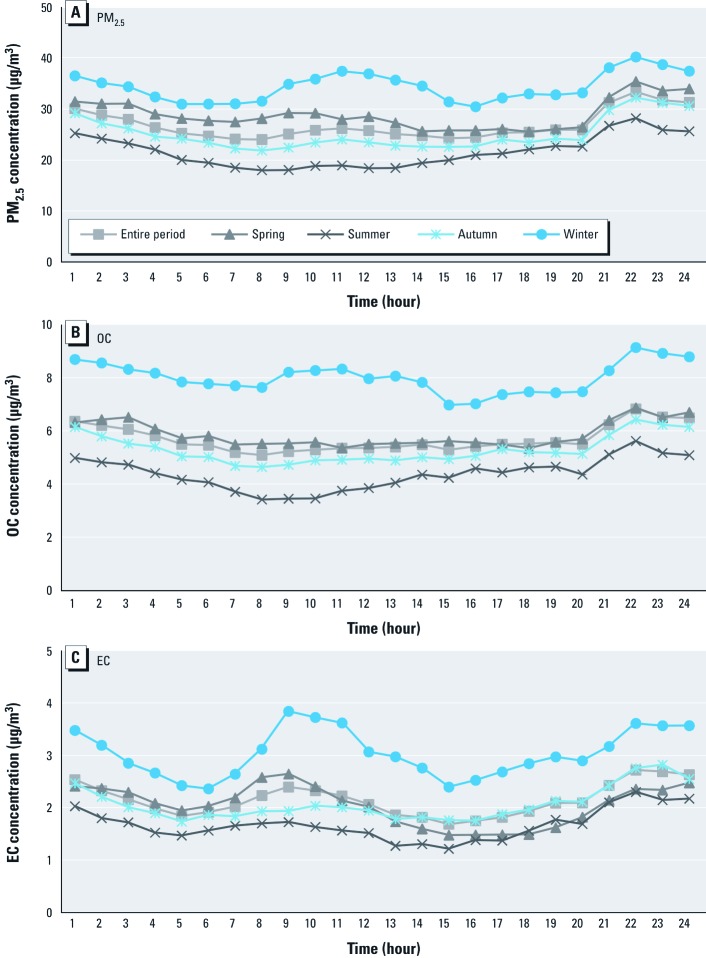
Daily pattern of hourly average of PM_2.5_ mass (*A*), OC (*B*), and EC (*C*) concentrations for the entire study period and by season.

Some components showed seasonal patterns. For instance, the mean concentrations of OC, EC, Cl, NO_3_, SO_4_, NH_4_, and K were 1.8, 1.8, 9.0, 2.1, 1.4, 1.7, and 4.0 times higher in winter than in summer, respectively. In contrast, Ca was 3.0 times higher in summer than in winter. Other components such as Na and Mg did not show distinct seasonal patterns. Supplemental Material, Figure 2 (http://dx.doi.org/10.1289/ehp.1104316) shows the percentage contribution of each PM_2.5_ component to PM_2.5_ total mass for the study period and by season. OC is the largest contributor for the entire period and for each season. For the entire study period and each season, the components OC, NO_3_, SO_4_, NH_4_, and EC comprise the majority (66–77%) of PM_2.5_ total mass. The relative contributions of the other five measured components (Cl, Ca, Mg, K, and Na) ranged from 0.3 to 1.2% of PM_2.5_ total mass for the study period, with no individual component accounting for more than 2.6% of the total mass in any season. The unmeasured chemical components representing the unidentifiable fraction accounted for 23.6% of the PM_2.5_ total mass for the study period, and from 19.3% to 30.7% for each individual season.

**Figure 2:**
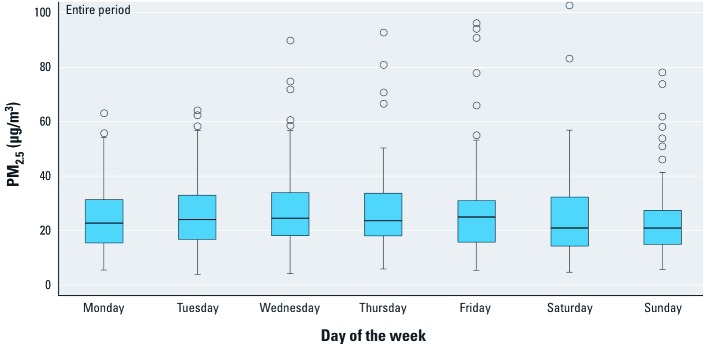
Boxplot of PM_2.5_ total mass by day of the week for the entire study period (August 2008 through October 2009). The boxes represent the IQR (25th–75th percentile); the horizontal line inside the box represents the median; the whiskers extend to the most extreme data point that is 1.5 times the IQR from the box; outlier values are shown as circles.

Figure 1 shows the daily pattern of pollutants by demonstrating trends of hourly averages of PM_2.5_ mass and some major components (OC and EC) for the entire period and each season. Results for other components are presented in Supplemental Material, Figure 3 (http://dx.doi.org/10.1289/ehp.1104316). PM_2.5_ mass concentrations displayed a peak in the evening (2200 hours) and a secondary peak in the morning (1100 hours). These peaks started to form around 0800 or 2000 hours when the heavy traffic usually occurred and increased several hours later. The evening peak is present for all seasons. Some other components (e.g., OC, EC, and NO_3_) also exhibit similar patterns of daily concentrations, with peaks in late morning and evening.

**Figure 3:**
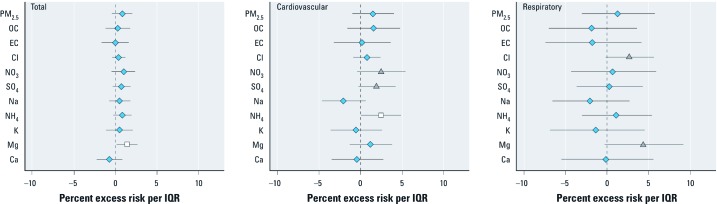
Percent change in risk for total (*A*), cardiovascular (*B*), and respiratory (*C*) mortality per IQR increase in PM_2.5_ mass and chemical components. Points represent central estimates, and horizontal lines represent 95% CIs.

Figure 2 shows the pattern of PM_2.5_ mass by day of the week for the entire period. Results by season are presented in Supplemental Material, Figure 4 (http://dx.doi.org/10.1289/ehp.1104316). Overall, we found similar mean levels by day of the week with higher concentrations on weekdays (27.4 ± 16.6 μg/m^3^) than on weekends (24.4 ± 16.2 μg/m^3^). Winter levels were higher than summer levels for each day of the week.

**Figure 4:**
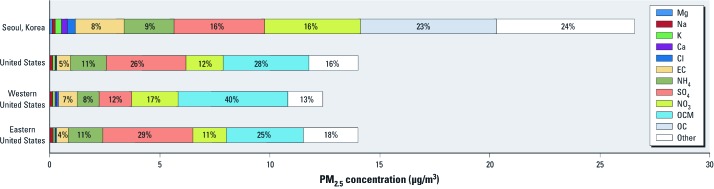
Percentage of PM_2.5_ total mass for each component, by location. OC is shown for Korea and OCM for the United States.

Table 2 provides correlations among PM_2.5_ mass and components. PM_2.5_ mass was strongly correlated with the major components of OC, EC, NO_3_, SO_4_, and NH_4_ (correlation, 0.85–0.94). PM_2.5_ mass was far less correlated with the components that contributed less to total mass. Correlations for PM_2.5_ and the components Cl, Na, K, Mg, and Ca ranged from –0.06 to 0.63. The major components were highly correlated with each other (range, 0.70–0.94). Correlations between daytime and 24-hr exposure for PM_2.5_ mass and each component were high [0.93–0.98; see Supplemental Material, Table 1 (http://dx.doi.org/10.1289/ehp.1104316)]. Correlations among PM_2.5_ mass and components by season are provided in the Supplemental Material, Table 2. PM_2.5_ was most strongly correlated with NH_4_ in all seasons (0.94–0.99).

**Table 2:** Correlation coefficients between PM_2.5_ and chemical components.

OC	EC	Cl	NO3	SO4	Na	NH4	K	Mg	Ca
PM2.5		0.91		0.85		0.45		0.90		0.89		–0.06		0.94		0.63		–0.05		–0.06
OC				0.91		0.57		0.81		0.75		0.01		0.81		0.69		–0.02		–0.06
EC						0.58		0.76		0.70		0.00		0.76		0.65		–0.01		–0.04
Cl								0.51		0.23		–0.04		0.39		0.69		–0.11		–0.21
NO3										0.72		–0.07		0.89		0.60		–0.13		–0.11
SO4												–0.01		0.94		0.46		–0.01		–0.08
Na														–0.07		0.13		–0.01		0.06
NH4																0.53		–0.07		–0.15
K																		–0.18		–0.16
Mg																				0.37


During the study period (August 2008 through October 2009), Asian dust storms occurred for 9 days (KMA 2011). We compared the levels of PM_2.5_ total mass and chemical components on dust storm and nondust storm days [see Supplemental Material, Table 3 (http://dx.doi.org/10.1289/ehp.1104316)]. For example, on days with dust events, overall PM_2.5_ levels were 58% higher (41.5 vs. 26.3 μg/m^3^), and levels of K and Ca were 2.5 times higher (0.5 vs. 0.2 μg/m^3^ for K, 0.5 vs. 0.2 for Ca). On dust days, the average percentage of PM_2.5_ total mass was 33% higher for K (1.2 vs. 0.9%) and 42% higher for Ca (1.7 vs. 1.2%) than on non-Asian dust days.

**Table 3:** Summary statistics of daily mortality and weather variables in Seoul, Korea (August 2008 through October 2009).

Mean	SD	Min	Max	IQR
Mortality (observations/day)		Total		92.0		11.4		64		138		16
		Cardiovascular		22.4		5.5		9		42		7
		Respiratory		5.4		2.3		1		13		3
Weather		Temperature (°C)		14.8		9.7		–9.2		30.0		16.6
		Relative humidity (%)		61.2		14.0		28.3		93.7		19.2
Abbreviations: Max, maximum; Min, minimum.												

*Associations between PM_2.5_ chemical components and mortality.* Table 3 shows summary statistics of daily cause-specific mortality and weather variables during the study period. The study included 42,022 deaths, with an average of 92 deaths/day, with 24% (22.4 deaths/day) from cardiovascular disease and 5.9% (5.4 deaths/day) from respiratory disease.

Supplemental Material, Table 4 (http://dx.doi.org/10.1289/ehp.1104316) shows the percentage change in risk of mortality per IQR increase in PM_2.5_ mass by lag. We identified the lag with the most certain effect estimates (largest *t*-statistics) for use in subsequent analysis of PM_2.5_ chemical components. These lags were the previous day (lag 1) for total and respiratory mortality and same day (lag 0) for cardiovascular mortality. An IQR increase in the previous day PM_2.5_ mass (16 μg/m^3^) was associated with a 0.80% (95% CI: –0.37%, 1.98%) increase in total mortality and a 1.25% (95% CI: –3.03%, 5.72%) increase in mortality from respiratory causes. An IQR increase in same-day PM_2.5_ was associated with a 1.47% (95% CI: –1.01%, 4.00%) increase in cardiovascular mortality.

Adjusted associations of IQR increases in PM_2.5_ chemical component concentrations on the previous day with total and respiratory mortality, and of concentrations on the same day with cardiovascular mortality are shown in Figure 3 and Supplemental Material, Table 5 (http://dx.doi.org/10.1289/ehp.1104316). For total mortality, all central estimates were positive except for EC and Ca. An IQR increase in Mg (0.05 μg/m^3^) was associated with a 1.4% increase (95% CI: 0.2%, 2.6%) in total mortality. For cardiovascular mortality, NO_3_, NH_4,_ and SO_4_ showed positive associations with cardiovascular mortality at the *p* < 0.10 level. These three components were correlated with each other and PM_2.5_ total mass (correlation 0.72 to 0.94). Cl and Mg also showed positive associations with respiratory mortality at the *p* < 0.10 level. Effect estimates based on daytime exposures were consistent with effect estimates based on 24-hr exposures (see Supplemental Material, Table 5).

Few data for NH_4_ were BDL (0.6% of the hourly observations); however a large fraction of Mg values were BDL (28.6%). The results shown in Figure 3 and in Supplemental Material, Table 5 (http://dx.doi.org/10.1289/ehp.1104316) are based on data for which hourly BDL values were replaced by half of the detection limit value. We conducted two sensitivity analyses for the main results for all components with any hourly BDL measurement: *a*) using actual recorded values, and *b*) omitting hourly BDL values. The effect estimates were generally consistent in terms of magnitude and direction in both cases (results not shown).

## Discussion

We characterized the PM_2.5_ mass and its chemical composition and estimated the cause-specific mortality effects of PM_2.5_ mass and its constituents in Seoul, Korea. We examined the levels of PM_2.5_ mass and its components, contribution of each component to total PM_2.5_ mass, and correlations among different components, including analysis by hour, day, and season. The degree of temporal variability by day or season differed by component. We performed, to the best of our knowledge, the first study of the relationship between chemical composition of particles and mortality in Asia, and observed significant positive associations between several components (e.g., Mg, NH_4_, and NO_3_) and cause-specific mortality in Seoul, Korea. For example, an IQR increase in previous-day Mg was associated with total and respiratory mortality. An IQR increase in same-day NH_4_ was associated with cardiovascular mortality.

The PM_2.5_ mass concentration was higher in winter and lower in summer; a similar finding was observed by Kim et al. (2007) in their study of PM_10_ and PM_2.5_ total mass in subways in the Seoul Metropolitan Subway station. The higher PM_2.5_ concentrations in winter are likely due to increased emissions (from combustion sources for heating) and a lower mixing height. The lower concentrations of PM_2.5_ in summer may relate to the large amounts of wet deposition as a function of precipitation, which is the major process of particle removal from the atmosphere, with 49% of the year’s deposition in this season (Kim et al. 2007). This seasonal pattern in PM_2.5_ levels is similar to that of the western United States, which has higher levels in winter, but the pattern is reversed in the eastern United States, which has higher levels in summer (Bell et al. 2007).

PM_2.5_ mass and some components (e.g., OC, EC, NO_3_, and NH_4_) showed similar patterns of daily concentrations, with peaks in late morning and evening. The peak started to form in the early morning or evening, attributed to the heavy traffic and increased several hours later. This trend has been observed in other regions. In North Carolina (USA), PM_2.5_ peaked in the early morning and midevening, and authors suggest that this pattern might be due to both exhaust of heavy traffic and diurnal evolution of planetary boundary layer (Aneja et al. 2006).

Our characterization of the chemical composition of PM_2.5_ mass identified several key components as major contributors (OC, 23.4%; NO_3_, 16.3%; SO_4_, 15.5%; EC, 8.3%; NH_4_, 8.6%). These components also constitute a substantial portion of PM_2.5_ total mass in the United States (organic carbon matter, 28%; SO_4_, 26%; NO_3_, 12%; NH_4_, 11%; EC, 5%) (Bell et al. 2007). Figure 4 shows the percentage of PM_2.5_ total mass for each component for Seoul, and for the continental United States and for the western and the eastern United States based on our previous work (Bell et al. 2007), which characterized spatial and temporal variability of PM_2.5_ components for 187 U.S. counties. Our results indicate that the PM_2.5_ in Seoul has a chemical structure that is more similar to the western United States than to the eastern United States with lower contributions of SO_4_ to PM_2.5_ at 16% for Seoul and 12% for the western United States, compared with 26% in the eastern United States, and higher contributions of NO_3_ at 16% for Seoul and 17% for the western United States compared with 11% for the eastern United States. The similarities between PM composition in Seoul and the western United States may relate to similar sources and transportation systems. For example, Seoul has several government policies to improve urban air quality, such as expanding the use of low-sulfur fuels and moving industrial sources out of the city (MOE 2008). SO_4_ concentrations in the eastern United States are typically higher than in the western United States because of the large number of coal-fired power plants in and upwind of the eastern United States. However, the overall level of PM_2.5_ in Seoul was 26.6 μg/m^3^, which exceeds the U.S. average of 14.0 μg/m^3^.

Local studies are needed because of variation in particle sources. For instance, we identified different chemical composition of particulate matter in Seoul during Asian dust storms. In Korea, Asian dust events are most frequent in spring and irregular during winter and fall (KMA 2011). Previous studies reported that the ambient air particles during Asian dust events were composed of particles of larger diameter than particles during non-dust periods (Chun et al. 2001; Kim et al. 2002). The potentially hazardous metal fractions of particles did not change during Asian dust events compared with usual conditions (Kim et al. 2003). Lee et al. (2007b) suggested that the mortality risks of urban air particles are likely to be underestimates if analysis is conducted during Asian dust events due to several factors such as differences in the chemical composition of particles during dust events with relatively less toxic components (e.g., increased larger particles, decreased hazardous metal fractions) and changes in indoor and outdoor behavioral patterns due to weather alerts or mass media announcements, which may reduce exposure to outdoor air pollution.

Our findings on the relative contribution of PM_2.5_ constituents in Seoul are consistent with previous studies of that region. Two studies reported that OC was the largest contributor to PM_2.5_ mass in Seoul (Heo et al. 2009; Kim et al. 2007). In Seoul, Korea, most of the OC originates from primary anthropogenic sources along with EC, of which major sources are combustion (e.g., gasoline, diesel vehicles) and biomass burning (Heo et al. 2009; Kim et al. 2007). EC originates primarily from combustion process and is associated with traffic-related sources (e.g., diesel emissions) in Seoul (Heo et al. 2009). In a Korean study, Park and Kim (2005) reported that motor vehicle exhaust (26%) was the major contributor to PM_2.5_ mass, which is related to OC and EC, followed by secondary sulfate (23%, SO_4_ and NH_4_) and nitrate (16%, NO_3_ and NH_4_), refuse incineration (15%, copper and zinc), soil dust [13%, Mg, aluminum (Al), and Ca], field burning (4%, K, OC, and EC), and oil combustion (2.7%, vanadium and nickel). In another study, the major contributors of PM_2.5_ in Korea were secondary nitrate including ammonium nitrate (20.9%, NO_3_ and NH_4_), secondary sulfate including ammonium sulfate (20.5%, SO_4_ and NH_4_), gasoline-fueled vehicles (17.2%, OC and EC), and biomass burning (12.1%, OC, EC, and K), with lesser contributions from diesel emissions (8.1%, EC and Ca), soil (7.4%, Mg, Al, Ca, and K), industry (6.7%, EC and Mn), road salt and two-stroke vehicles (5.1%, OC, NO_3_, Cl, and Na), and aged sea salt (2.2%, Na, Mg, K, and OC) (Heo et al. 2009). With additional information on source profiles for this region, future research could use PM_2.5_ chemical component data with approaches such as source factorization to link health risk to specific sources, as has been done in other areas (Bell et al. 2010; Halonen et al. 2009; Ostro et al. 2011).

We found a significant positive association between Mg and total mortality. Bae et al. (2010) found a significant positive association between PM_2.5_-bound Mg and an oxidative stress biomarker (urinary malondialdehyde). Secondary products of fuel combustion (NO_3_, SO_4_, and NH_4_) also exhibited the stronger associations with cardiovascular mortality than did other components. However, it is difficult to identify individual effects of PM_2.5_ components because every component has multiple and shared sources and effects observed for one component may be the result of a component with similar sources. The observed associations for fuel combustion components may be acting as a marker of other components from similar sources. For example, although OC and EC can result from combustion of fossil fuel, biomass burning may also produce these components (Heo et al. 2009; Park and Kim 2005).

We found a higher central estimate of PM_2.5_ mass for cardiovascular mortality than for respiratory mortality, although neither association was statistically significant. Several components that were among the largest contributors to PM_2.5_ total mass (NO_3_, SO_4_, and NH_4_) were moderately associated with cardiovascular mortality (*p* < 0.10). Other components with smaller mass contributions (Mg and Cl) were moderately associated with respiratory mortality (*p* < 0.10). Previous findings for the associations between specific PM components and health risk have been inconsistent (Bell et al. 2009; Franklin et al. 2008; Laden et al. 2000; Peng et al. 2009; Zhou et al. 2011). Ostro et al. (2007) examined associations between PM_2.5_ mass and its components and daily mortality in six California counties from 2000 to 2003 and found that PM_2.5_ mass and several constituents such as EC, OC, and NO_3_ were associated with multiple mortality categories, especially cardiovascular deaths. In a study in Santa Clara County, California (USA), Fairley (2003) reported that NO_3_ was associated with cardiovascular mortality as well as all-cause mortality. Brook et al. (2004) suggested possible biological mechanisms linking PM exposures with cardiovascular disease (e.g., direct effects of pollutants on the cardiovascular system, blood, and lung receptors, indirect effects mediated through pulmonary oxidative stress and inflammatory responses). Ostro et al. (2008) found that cardiovascular mortality in six California counties was associated with PM_2.5_ and several PM_2.5_ components including EC, OC, nitrates, sulfates, K, copper, and iron. In a study in Phoenix, Arizona (USA), Mar et al. (2000) reported that EC, OC, and K were associated with mortality. These various results may be due to the diversity of the study regions, different pollutant mixtures, and health outcomes (Zhou et al. 2011) and demonstrate the need for region-specific studies.

Limitations of this study include the use of a single monitoring site, which may introduce errors in exposure measurements and lack of representativeness for exposures to certain local sources (e.g., traffic) in the analysis of components and mortality. The spatial heterogeneity of concentrations varies by PM_2.5_ chemical component (Bell et al. 2011; Peng and Bell 2010); therefore, future work would benefit from multiple monitors within a given community. Detection limits can limit analysis of PM components at low levels. Measurement error may also cause estimates of some components to be more accurate than others (Bell et al. 2009, 2011). Another limitation is the relatively shorter study period compared with previous studies. However, the actual number of observations in our study was large because we used consecutive hourly data for the entire study period. Future research with a longer time frame of PM component data is warranted.

Advantages of this study include the availability of hourly data on PM_2.5_ and PM_2.5_ chemical composition. We were able to characterize temporal patterns in PM composition by hour, day, and season and to estimate daytime exposure effects using hourly data and cumulative short-term exposure effects with consecutive daily data. Most of the previous studies in the United States used chemical speciation data every third day (e.g., Ito et al. 2011).

In conclusion, we found temporal variation of PM_2.5_ mass and chemical components and identified associations between specific PM_2.5_ components and cause-specific mortality, particularly Mg and NH_4_. Our findings have implications for epidemiologic research on PM_2.5_ characteristics and provide evidence that links PM_2.5_ and its constituents with mortality in Korea.

## Supplemental Material

(545 KB) PDF
