# Supplementary material for: Characterization of Fine Particulate Matter and Associations between Particulate Chemical Constituents and Mortality in Seoul, Korea
Source: Environ Health Perspect. 2012 Mar 22;120(6):872–8. doi: 10.1289/ehp.1104316 (PMC3385433; doi:10.1289/ehp.1104316)
Supplement: (545 KB) PDF [file ehp.1104316.s001.pdf]

Supplemental Materials

Characterization of Fine Particulate Matter and Associations between Particulate Chemical Constituents and Mortality in Seoul, Korea

Ji-Young Son, Jong-Tae Lee, Ki-Hyun Kim, Kweon Jung, Michelle L. Bell

Table of Contents

Figures

Figure 1. Comparison of concentrations of daytime and 24-hour average for $PM_{2.5}$ mass and each component.

Figure 2. Contribution of components to $PM_{2.5}$ for the study period and by season.

Figure 3. Daily pattern of hourly average of $PM_{2.5}$ mass and other component concentrations for the study period and by season.

Figure 4. Boxplots of $PM_{2.5}$ total mass by day of the week by season.

Tables

Table 1. Correlations coefficients between 24-hour and daytime $PM_{2.5}$ and chemical components

Table 2. Correlations coefficients between 24-hour average $PM_{2.5}$ and chemical component levels by season

Table 3. Levels of $PM_{2.5}$ total mass and chemical components on dust storm and non-dust storm days

Table 4. Percent change (95% confidence intervals) in mortality risk associated with an IQR increase in $PM_{2.5}$ by lag

Table 5. Percent change (95% confidence intervals) in mortality risk per IQR increase in $PM_{2.5}$ chemical components

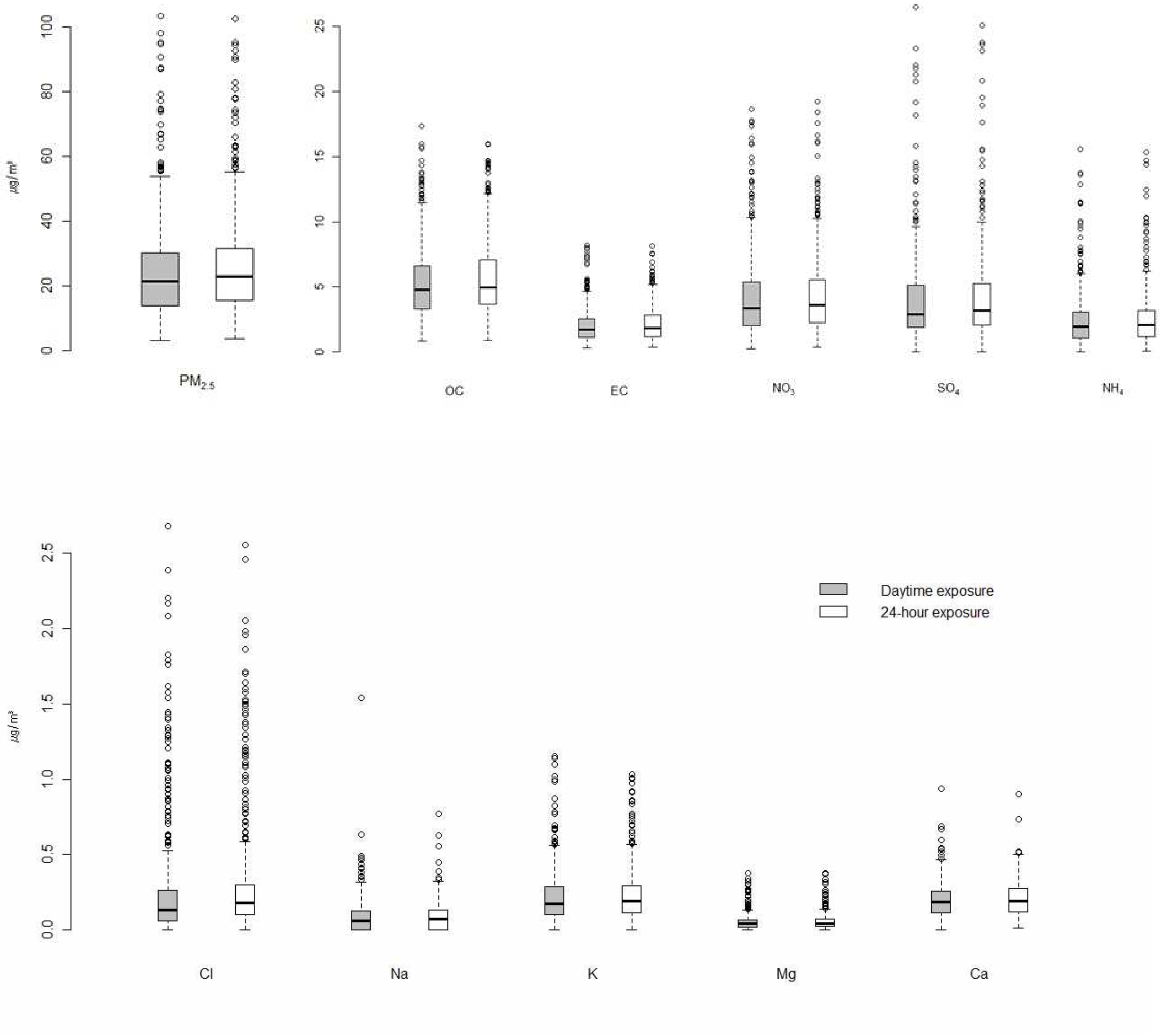
μg/m³
0
20
40
60
80
100
0
5
10
15
20
25
PM2.5
OC
EC
NO3
SO4
NH4
μg/m³
0.0
0.5
1.0
1.5
2.0
2.5
Daytime exposure
24-hour exposure
Cl
Na
K
Mg
Ca

Supplemental Material, Figure 1. Comparison of concentrations of daytime and 24-hour average for $PM_{2.5}$ mass and each component.

Note: Daytime exposure corresponds to exposure from 8am to 8pm. $PM_{2.5}$ total mass and $PM_{2.5}$ components have different scales for the y-axis. Boxes extend from the 25th to the 75th percentile, horizontal bars represent the median, whiskers extend to the most extreme data point that is 1.5 times interquartile range (IQR) from the box, and outliers are represented as points.

Supplemental Material, Figure 2. Contribution of components to $PM_{2.5}$ for the study period and by season.

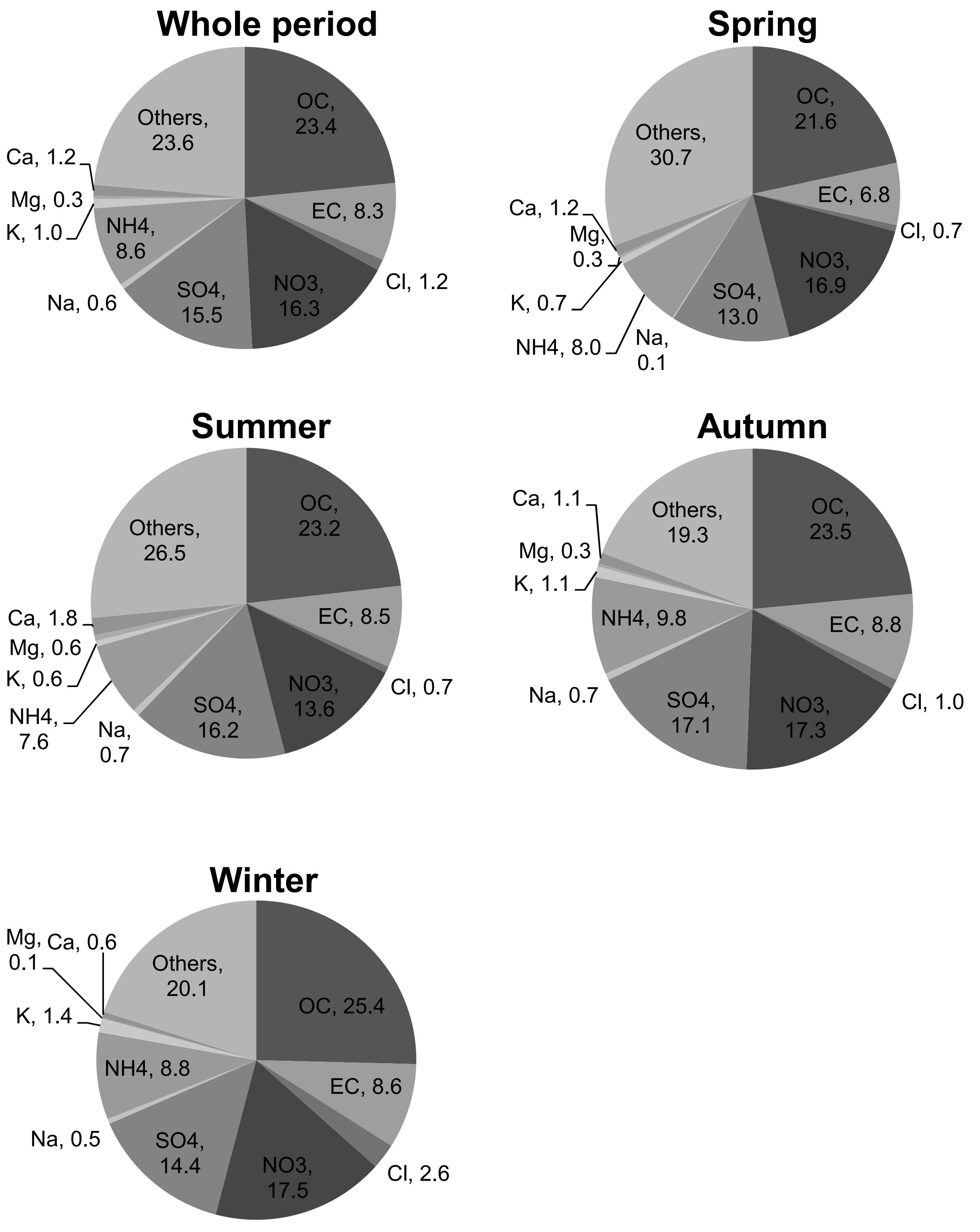

Supplemental Material, Figure 3. Daily pattern of hourly average of $PM_{2.5}$ mass and other component concentrations for the study period and by season.

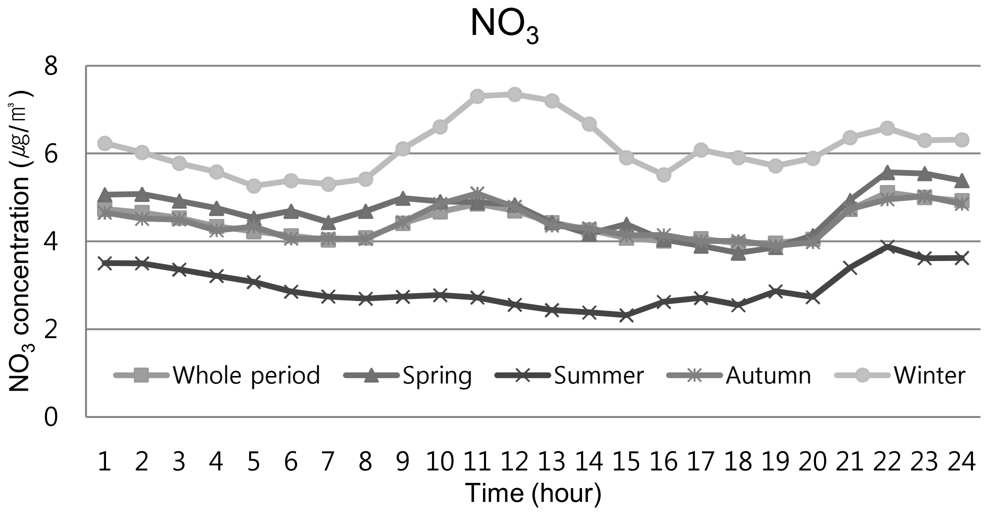

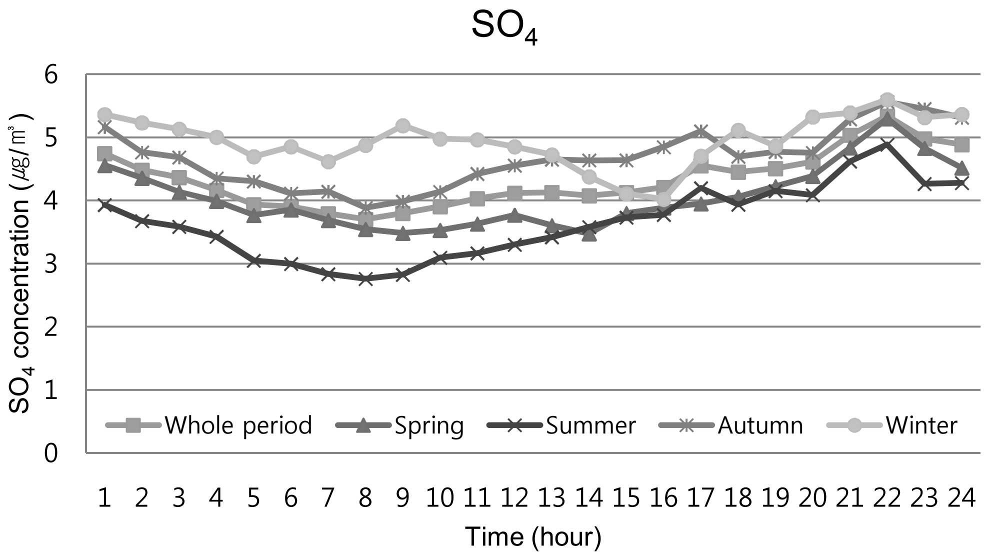

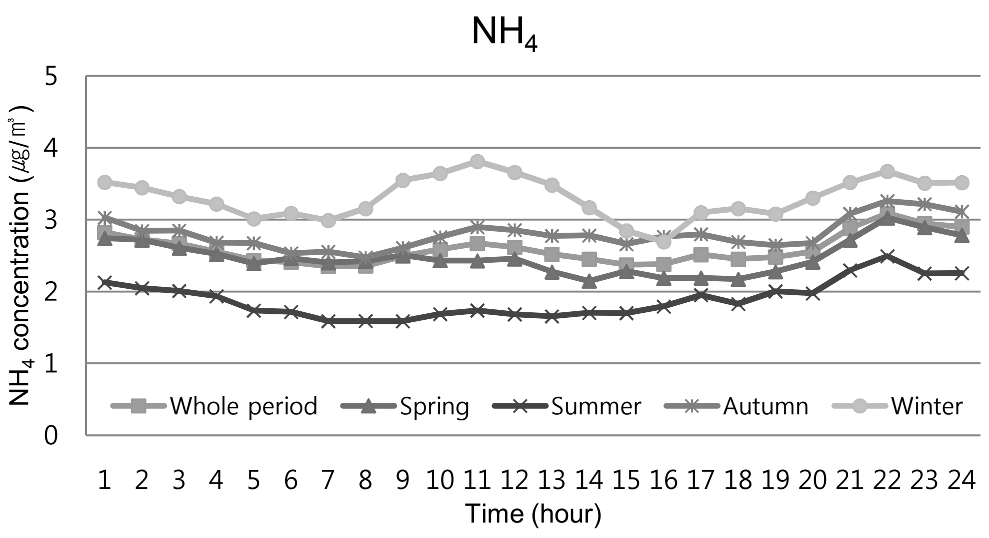

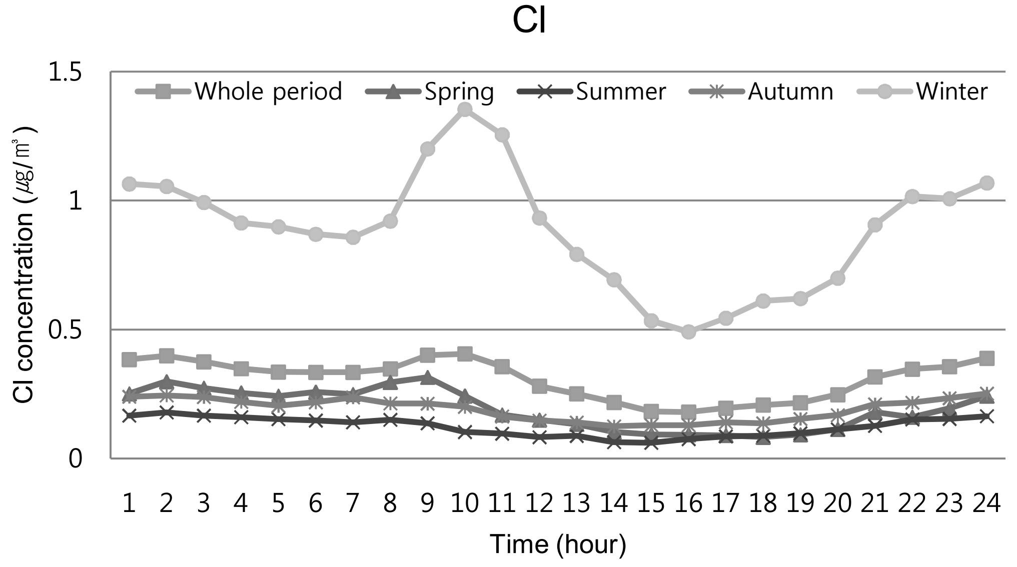
Cl
1.5
1
0.5
0
Whole period
Spring
Summer
Autumn
Winter
Cl concentration (㎍/㎥)
1 2 3 4 5 6 7 8 9 10 11 12 13 14 15 16 17 18 19 20 21 22 23 24
Time (hour)

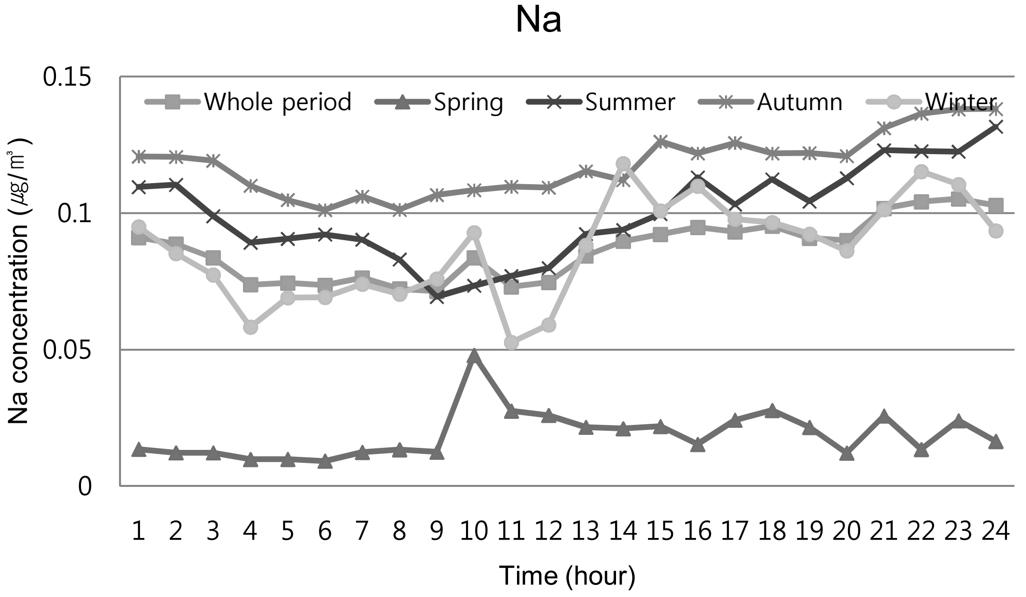
Na
0.15
0.1
0.05
0
Whole period
Spring
Summer
Autumn
Winter
Na concentration (㎍/㎥)
1 2 3 4 5 6 7 8 9 10 11 12 13 14 15 16 17 18 19 20 21 22 23 24
Time (hour)

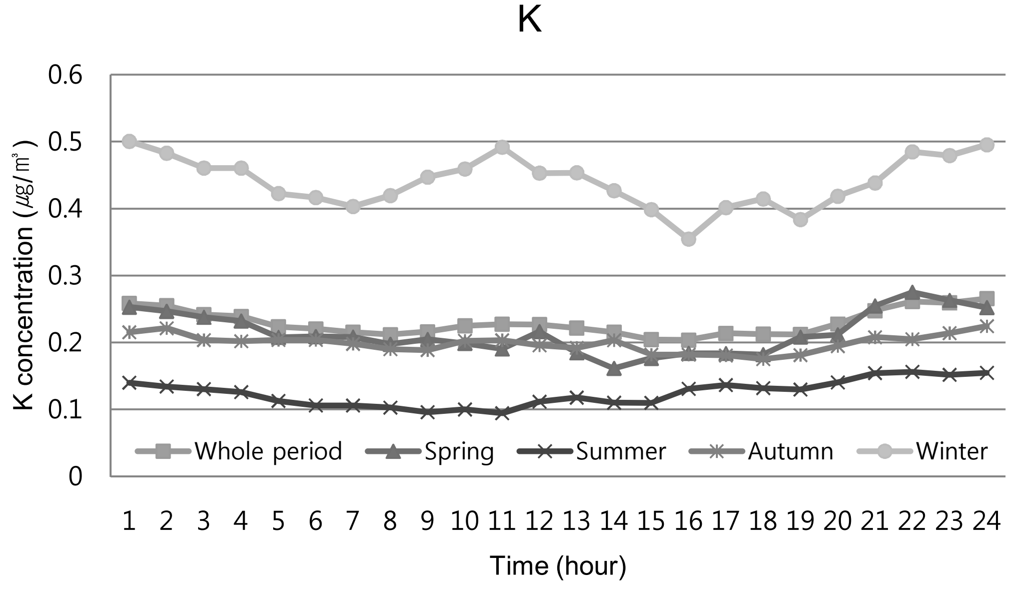
K
0.6
0.5
0.4
0.3
0.2
0.1
0
Whole period
Spring
Summer
Autumn
Winter
K concentration (㎍/㎥)
1 2 3 4 5 6 7 8 9 10 11 12 13 14 15 16 17 18 19 20 21 22 23 24
Time (hour)

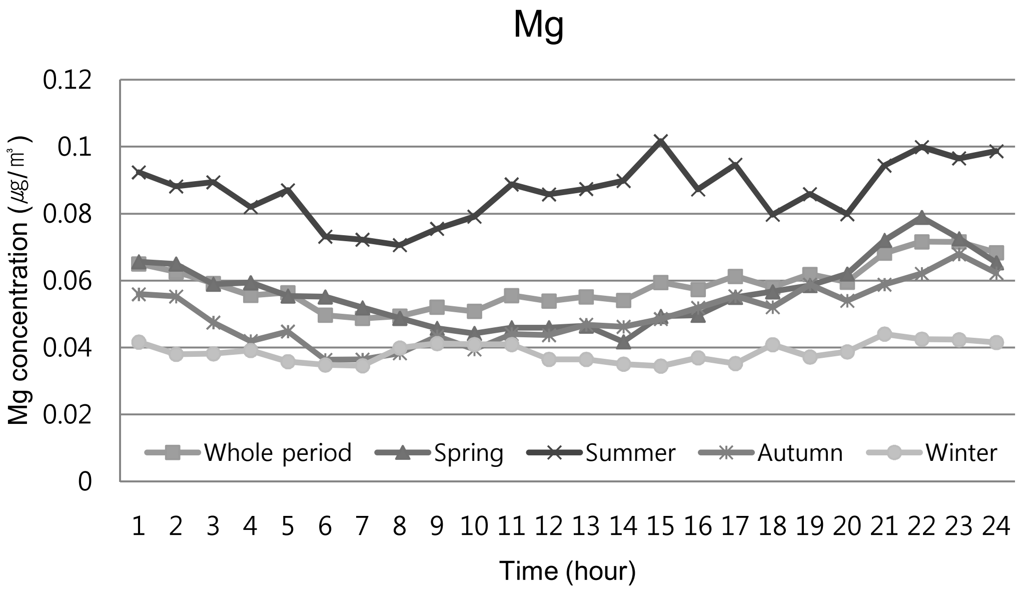
Mg
0.12
0.1
0.08
0.06
0.04
0.02
0
Mg concentration (㎍/㎥)
Whole period
Spring
Summer
Autumn
Winter
1 2 3 4 5 6 7 8 9 10 11 12 13 14 15 16 17 18 19 20 21 22 23 24
Time (hour)

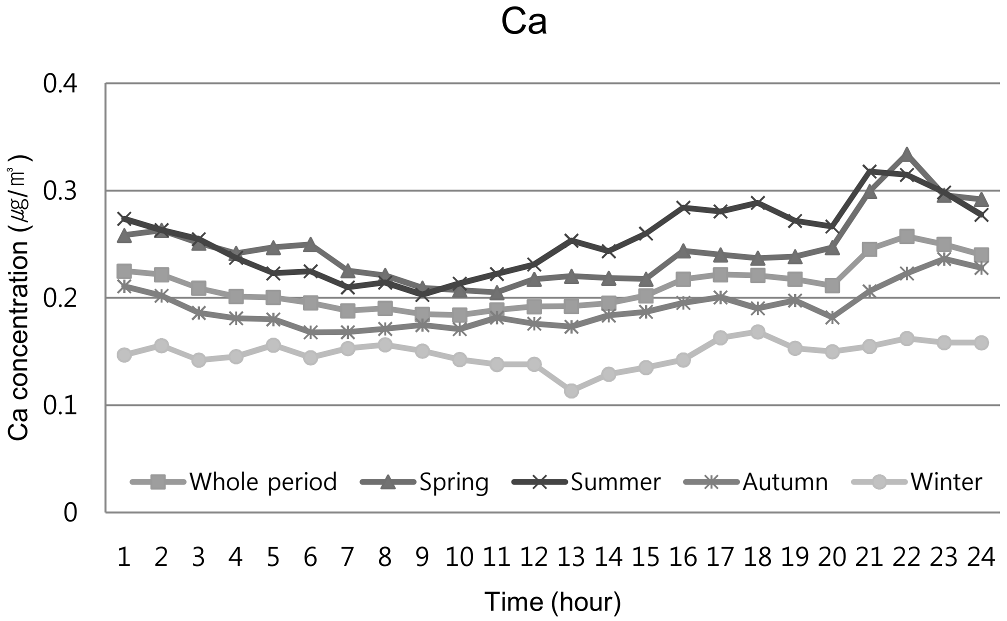
Ca
0.4
0.3
0.2
0.1
0
Ca concentration (㎍/㎥)
Whole period
Spring
Summer
Autumn
Winter
1 2 3 4 5 6 7 8 9 10 11 12 13 14 15 16 17 18 19 20 21 22 23 24
Time (hour)

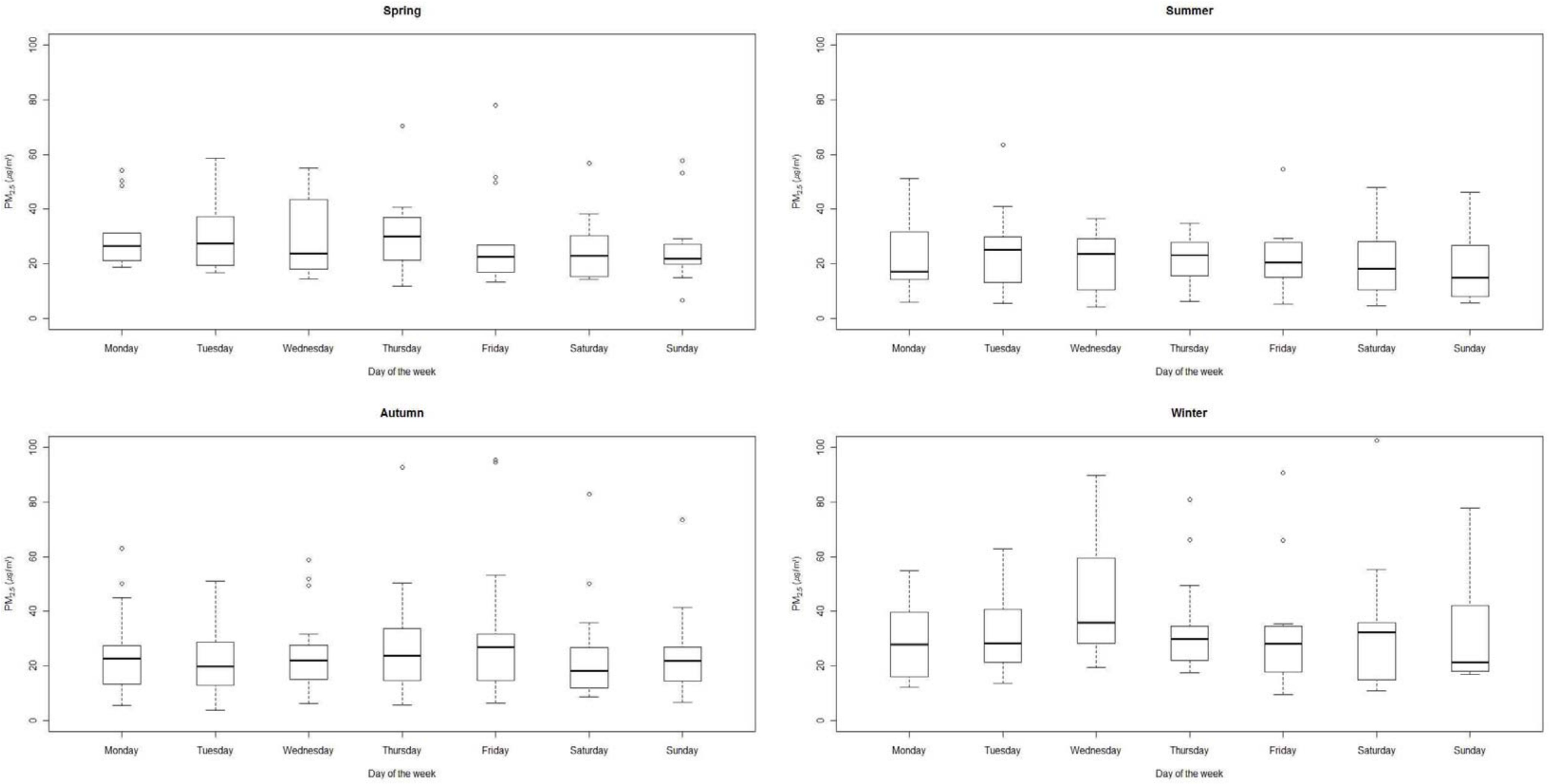

Supplemental Material, Figure 4. Boxplots of $PM_{2.5}$ total mass by day of the week by season.

Note: Boxes extend from the 25th to the 75th percentile, horizontal bars represent the median, whiskers extend to the most extreme data point that is 1.5 times interquartile range (IQR) from the box, and outliers are represented as points.

Supplemental Material, Table 1. Correlations coefficients between 24-hour and daytime $PM_{2.5}$ and chemical components

		Daytime exposure										
		$PM_{2.5}$	OC	EC	Cl	NO_3	SO_4	Na	NH_4	K	Mg	Ca
24-hour Exposure	$PM_{2.5}$	**0.98**	0.89	0.83	0.46	0.87	0.87	-0.05	0.93	0.62	-0.03	-0.06
	OC	0.89	**0.97**	0.89	0.56	0.80	0.74	0.01	0.81	0.67	-0.01	-0.06
	EC	0.83	0.89	**0.95**	0.56	0.76	0.68	-0.02	0.76	0.62	-0.00	-0.04
	Cl	0.46	0.57	0.61	**0.96**	0.56	0.22	-0.04	0.40	0.66	-0.09	-0.19
	NO_3	0.87	0.80	0.76	0.52	**0.97**	0.70	-0.06	0.87	0.58	-0.12	-0.10
	SO_4	0.86	0.74	0.69	0.25	0.68	**0.98**	-0.01	0.93	0.47	0.01	-0.06
	Na	-0.06	0.02	0.02	-0.05	-0.06	0.01	**0.93**	-0.06	0.15	0.04	0.11
	NH_4	0.91	0.80	0.75	0.41	0.85	0.92	-0.06	**0.98**	0.53	-0.05	-0.13
	K	0.63	0.69	0.68	0.67	0.63	0.46	0.10	0.55	**0.98**	-0.15	-0.15
	Mg	-0.04	-0.00	-0.02	-0.11	-0.13	0.01	-0.02	-0.06	-0.16	**0.97**	0.34
	Ca	-0.06	-0.06	-0.07	-0.21	-0.12	-0.07	0.03	-0.15	-0.15	0.35	**0.95**
Daytime exposure	$PM_{2.5}$		0.91	0.85	0.49	0.87	0.86	-0.04	0.92	0.63	-0.01	-0.06
	OC			0.91	0.58	0.82	0.76	0.03	0.83	0.68	0.02	-0.04
	EC				0.63	0.79	0.70	0.00	0.79	0.66	-0.00	-0.05
	Cl					0.58	0.25	-0.03	0.43	0.67	-0.08	-0.18
	NO_3						0.68	-0.04	0.88	0.62	-0.11	-0.11
	SO_4							0.02	0.93	0.48	0.04	-0.04
	Na								-0.05	0.16	0.03	0.13
	NH_4									0.55	-0.03	-0.13
	K										-0.13	-0.11
	Mg											0.34

Supplemental Material, Table 2. Correlations coefficients between 24-hour average $PM_{2.5}$ and chemical component levels by season

	OC	EC	Cl	NO_3	SO_4	Na	NH_4	K	Mg	Ca
Spring										
$PM_{2.5}$	0.92	0.91	0.16	0.91	0.95	-0.10	0.99	0.81	-0.18	-0.32
OC		0.86	0.22	0.87	0.81	-0.05	0.89	0.86	-0.16	-0.29
EC			0.18	0.86	0.82	-0.06	0.88	0.74	-0.21	-0.29
Cl				0.30	-0.02	-0.00	0.15	0.20	-0.05	-0.07
NO_3					0.76	-0.08	0.90	0.74	-0.16	-0.33
SO_4						-0.13	0.95	0.74	-0.12	-0.27
Na							-0.14	-0.01	-0.01	0.32
NH_4								0.78	-0.22	-0.39
K									0.05	-0.20
Mg										0.55
Summer										
$PM_{2.5}$	0.85	0.84	0.59	0.81	0.86	0.11	0.94	0.60	0.03	0.30
OC		0.89	0.48	0.63	0.72	0.22	0.78	0.57	0.15	0.31
EC			0.52	0.60	0.74	0.14	0.79	0.48	0.17	0.25
Cl				0.52	0.47	0.12	0.59	0.32	0.20	0.07
NO_3					0.43	0.18	0.70	0.62	-0.19	0.24
SO_4						-0.05	0.91	0.37	0.18	0.24
Na							-0.08	0.58	-0.03	0.10
NH_4								0.42	0.13	0.21
K									-0.17	0.35
Mg										0.09
Autumn										
$PM_{2.5}$	0.94	0.81	0.52	0.91	0.93	0.15	0.97	0.34	-0.01	0.03
OC		0.93	0.42	0.80	0.84	0.10	0.86	0.30	0.07	0.07
EC			0.34	0.67	0.72	0.07	0.72	0.24	0.10	0.07
Cl				0.60	0.40	0.40	0.53	0.61	-0.08	-0.00
NO_3					0.75	0.11	0.89	0.28	0.03	0.07
SO_4						0.20	0.96	0.38	-0.12	-0.05

Table 2 (continued)

	OC	EC	Cl	NO_3	SO_4	Na	NH_4	K	Mg	Ca
Na							0.17	0.33	0.02	0.00
NH_4								0.38	-0.14	-0.07
K									-0.18	-0.03
Mg										0.71
Winter										
$PM_{2.5}$	0.90	0.85	0.57	0.93	0.91	-0.23	0.96	0.75	0.34	0.05
OC		0.92	0.69	0.85	0.74	-0.07	0.81	0.77	0.30	0.15
EC			0.77	0.86	0.68	-0.10	0.79	0.75	0.24	0.24
Cl				0.66	0.34	-0.21	0.56	0.55	0.24	-0.01
NO_3					0.82	-0.33	0.95	0.71	0.26	-0.04
SO_4						-0.24	0.94	0.69	0.35	-0.00
Na							-0.37	0.04	-0.02	0.21
NH_4								0.71	0.33	-0.06
K									0.30	0.04
Mg										0.39

Supplemental Material, Table 3. Levels of $PM_{2.5}$ total mass and chemical components on dust storm and non-dust storm days

	Mass of component ($\mu g/m^3$)			Average percent of $PM_{2.5}$ total mass (IQR of percents)
	Average ± SD	Minimum-Maximum	IQR	
During the Asian dust days (N= 9)				
$PM_{2.5}$	41.5 ± 19.6	15.0-65.8	37.4	-
OC	8.5 ± 4.3	3.3-14.6	6.9	22.3 (4.4)
EC	3.6 ± 2.6	0.7-8.2	2.4	8.5 (5.8)
Cl	0.6 ± 0.5	0.0-1.5	0.5	1.3 (1.2)
NO_3	6.3 ± 2.8	1.9-10.6	4.1	16.5 (4.0)
SO_4	4.9 ± 2.5	1.3-7.2	4.9	12.6 (1.4)
Na	0.1 ± 0.1	0.0-0.3	0.2	0.4 (0.5)
NH_4	3.0 ± 1.9	0.6-5.2	3.4	7.0 (3.7)
K	0.5 ± 0.3	0.1-0.9	0.5	1.2 (0.6)
Mg	0.1 ± 0.0	0.0-0.2	0.0	0.3 (0.1)
Ca	0.5 ± 0.2	0.2-0.9	0.5	1.7 (0.6)
During the non-Asian dust days (N=448)				
$PM_{2.5}$	26.3 ± 16.3	3.7-102.4	15.7	-
OC	5.7 ± 2.9	0.9-16.0	3.3	23.4 (6.3)
EC	2.1 ± 1.3	0.4-7.5	1.7	8.3 (3.2)
Cl	0.3 ± 0.4	0.0-2.5	0.2	1.2 (1.1)
NO_3	4.4 ± 3.1	0.4-19.3	3.2	16.3 (6.7)
SO_4	4.3 ± 3.6	0.0-25.1	3.2	15.5 (6.9)
Na	0.1 ± 0.1	0.0-0.8	0.1	0.6 (0.7)
NH_4	2.6 ± 2.2	0.1-15.3	2.0	8.7 (2.7)
K	0.2 ± 0.2	0.0-1.0	0.2	0.9 (0.8)
Mg	0.1 ± 0.1	0.0-0.4	0.0	0.3 (0.3)
Ca	0.2 ± 0.1	0.0-0.5	0.1	1.2 (1.1)

Supplemental Material, Table 4. Percent change (95% confidence intervals) in mortality risk associated with an IQR increase in $PM_{2.5}$ by lag

Lag	Percentage change in risk of mortality					
	Total		Cardiovascular		Respiratory	
0	0.64	(-0.58, 1.87)	1.47	(-1.01, 4.00)	-1.95	(-6.34, 2.65)
1	0.80	(-0.37, 1.98)	0.11	(-2.24, 2.52)	1.25	(-3.03, 5.72)
2	-0.08	(-1.20, 1.05)	-0.44	(-2.70, 1.87)	-0.38	(-4.47, 3.89)
3	-0.26	(-1.35, 0.83)	0.36	(-1.82, 2.59)	-2.25	(-6.16, 1.82)
0-1	0.90	(-0.44, 2.27)	0.97	(-1.75, 3.76)	-0.42	(-5.29, 4.69)
0-2	0.65	(-0.80, 2.12)	0.52	(-2.41, 3.53)	-0.52	(-5.77, 5.03)
0-3	0.43	(-1.12, 2.00)	0.71	(-2.40, 3.91)	-1.69	(-7.22, 4.18)

Note: IQR for $PM_{2.5}$ is 16.0μg/m^3. The estimates were derived from over-dispersed Poisson generalized linear model, adjusting for time, day of week, temperature, and relative humidity.

Supplemental Material, Table 5. Percent change (95% confidence intervals) in mortality risk per IQR increase in $PM_{2.5}$ chemical components

	24-hour exposure						Daytime exposure (12-hour)					
Component	Total		Cardiovascular		Respiratory		Total		Cardiovascular		Respiratory	
OC	0.29	(-1.16, 1.75)	1.56	(-1.52, 4.73)	-1.86	(-6.97, 3.52)	0.33	(-1.03, 1.71)	1.33	(-1.60, 4.35)	-0.48	(-5.37, 4.65)
EC	-0.04	(-1.62, 1.56)	0.17	(-3.16, 3.60)	-1.80	(-7.38, 4.11)	-0.12	(-1.45, 1.23)	0.05	(-2.81, 2.99)	-1.59	(-6.37, 3.42)
Cl	0.38	(-0.38, 1.15)	0.76	(-0.83, 2.37)	2.67*	(-0.14, 5.57)	0.41	(-0.31, 1.14)	0.81	(-0.70, 2.35)	2.61*	(-0.03, 5.33)
NO_3	0.96	(-0.38, 2.31)	2.46*	(-0.38, 5.37)	0.65	(-4.30, 5.85)	0.84	(-0.49, 2.19)	2.66*	(-0.17, 5.56)	-0.20	(-5.13, 4.99)
SO_4	0.71	(-0.34, 1.77)	1.97*	(-0.24, 4.23)	0.23	(-3.66, 4.28)	0.63	(-0.40, 1.67)	2.03*	(-0.15, 4.25)	0.50	(-3.34, 4.49)
Na	0.48	(-0.76, 1.75)	-2.07	(-4.63, 0.55)	-2.05	(-6.54, 2.66)	0.68	(-0.27, 1.63)	-1.80*	(-3.85, 0.29)	-0.22	(-3.80, 3.51)
NH_4	0.83	(-0.27, 1.94)	2.44†	(0.12, 4.81)	1.10	(-3.01, 5.37)	0.72	(-0.36, 1.82)	2.58†	(0.28, 4.93)	0.73	(-3.33, 4.96)
K	0.49	(-1.06, 2.06)	-0.57	(-3.61, 2.56)	-1.36	(-6.86, 4.47)	0.81	(-0.71, 2.35)	0.34	(-2.67, 3.44)	-0.95	(-6.41, 4.82)
Mg	1.39†	(0.18, 2.61)	1.18	(-1.28, 3.70)	4.34*	(-0.26, 9.16)	1.28†	(0.08, 2.49)	0.86	(-1.59, 3.37)	5.07†	(0.47, 9.87)
Ca	-0.74	(-2.23, 0.77)	-0.45	(-3.48, 2.68)	-0.14	(-5.50, 5.53)	-0.34	(-1.30, 0.64)	-0.55	(-2.54, 1.47)	0.36	(-3.15, 4.00)

Note: Lag 1 for total and respiratory mortality; Lag 0 for cardiovascular mortality. Lag0 represents exposure on the same day, lag1 represents the exposure on the previous day. The estimates were derived from over-dispersed Poisson generalized linear model, adjusting for time, day of week, temperature, and relative humidity.

* $p < 0.10$ † $p < 0.05$
